# Can Stemness and Chemoresistance Be Therapeutically Targeted via Signaling Pathways in Ovarian Cancer?

**DOI:** 10.3390/cancers10080241

**Published:** 2018-07-24

**Authors:** Lynn Roy, Karen D. Cowden Dahl

**Affiliations:** 1 Harper Cancer Research Institute, South Bend, IN 46617, USA; lmroy@iupui.edu; 2 Department of Biochemistry and Molecular Biology, Indiana University School of Medicine-South Bend, South Bend, IN 46617, USA; 3 Department of Chemistry and Biochemistry, University of Notre Dame, Notre Dame, IN 46617, USA; 4 Indiana University Melvin and Bren Simon Cancer Center, Indianapolis, IN 46202, USA

**Keywords:** ovarian cancer, cancer stem cells, signaling, chemoresistance, metastasis

## Abstract

Ovarian cancer is the most lethal gynecological malignancy. Poor overall survival, particularly for patients with high grade serous (HGS) ovarian cancer, is often attributed to late stage at diagnosis and relapse following chemotherapy. HGS ovarian cancer is a heterogenous disease in that few genes are consistently mutated between patients. Additionally, HGS ovarian cancer is characterized by high genomic instability. For these reasons, personalized approaches may be necessary for effective treatment and cure. Understanding the molecular mechanisms that contribute to tumor metastasis and chemoresistance are essential to improve survival rates. One favored model for tumor metastasis and chemoresistance is the cancer stem cell (CSC) model. CSCs are cells with enhanced self-renewal properties that are enriched following chemotherapy. Elimination of this cell population is thought to be a mechanism to increase therapeutic response. Therefore, accurate identification of stem cell populations that are most clinically relevant is necessary. While many CSC identifiers (ALDH, OCT4, CD133, and side population) have been established, it is still not clear which population(s) will be most beneficial to target in patients. Therefore, there is a critical need to characterize CSCs with reliable markers and find their weaknesses that will make the CSCs amenable to therapy. Many signaling pathways are implicated for their roles in CSC initiation and maintenance. Therapeutically targeting pathways needed for CSC initiation or maintenance may be an effective way of treating HGS ovarian cancer patients. In conclusion, the prognosis for HGS ovarian cancer may be improved by combining CSC phenotyping with targeted therapies for pathways involved in CSC maintenance.

## 1. Introduction

In the United States, ovarian cancer is the fifth leading cause of cancer death in women [1]. The American Cancer Society (ACS) estimates that this year approximately 22,240 women will be newly diagnosed with ovarian cancer, and ~14,075 women will die as a result of the disease, making it the most lethal gynecologic malignancy (ACS Facts and Figures 2018). The vagueness of symptoms (bloating, abdominal/pelvic pain, difficulty eating/feeling of fullness, and frequent urination) and the lack of early detection methods contribute to the majority of patients (70–75%) receiving diagnoses in advanced stages (stage III or stage IV) when the cancer has metastasized throughout the peritoneal cavity [1,2]. The five-year survival rate for women with advanced-stage ovarian cancer is ~25% [3,4].

There are several major ovarian cancer subtypes. Additionally, there is mutational and gene expression heterogeneity within each subgenre. Mutational and gene expression heterogeneity is also found in different subpopulations within a single tumor. Patients with the same pathological diagnosis, such as high grade serous (HGS) carcinoma, often vary greatly with respect to gene expression and specific genetic mutations [3,5,6]. The lack of consistent mutations or mis-expressed genes makes developing novel targeted therapeutics difficult. The current standard of care is a “one size fits all” approach consisting of aggressive debulking surgery to resect visible tumor followed by platinum and taxane combination chemotherapy [1,7,8,9]. Residual tumor implants measuring less than 1 cm are considered indicative of optimal debulking [1]. Debulking surgery performed by a gynecological oncologist improves the chance of survival; however, many patients are not treated by gynecological oncologists [1,7,8]. Therefore, in some cases, chemotherapy prior to surgery is equally effective as primary debulking [4]. Chemotherapy treatment is initially effective in 70–80% of patients [2,10,11]. However, recurrence of the disease will occur in the majority of patients (80–90%) within 5 years, and the tumors often acquire resistance to the chemotherapeutics [1,9,11]. The presence of microscopic tumors left behind during surgical debulking and the limitations of current chemotherapeutics contribute to the likelihood of relapse. The presence or enrichment of cancer stem cells (CSCs), which are defined as tumor cells that survive and/or accumulate after chemotherapy, have activation of self-renewing signaling pathways, and exhibit increased tumor-initiating properties, may contribute to relapse [11,12,13]. We will discuss how CSC properties contribute to chemoresistance and how investigating these properties may lead to novel therapeutics to eliminate ovarian cancer and prevent relapse.

## 2. Histologic Types of Ovarian Cancer

**Figure 1:**
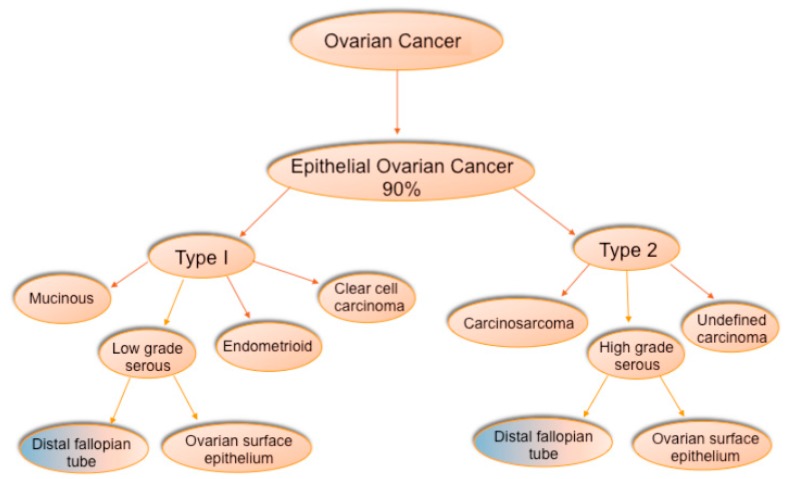
Classification of the Epithelial Ovarian Cancer histological subtype according to the two tier system. Type I tumors include endometroid, clear cell carcinoma, mucinous, and low grade serous. Type II tumors are mostly comprised of high grade serous but also include carcinosarcoma and undefined carcinomas [5,15,18,20].

Ovarian tumors are divided into three types: epithelial (60%), germ cell (30%), and specialized stromal cells tumors (8%) [3,14]. Epithelial tumors comprise the majority of malignant ovarian tumors (80–90%) [10,14]. Within the epithelial tumors there are four major subtypes: serous, endometrioid, clear cell, and mucinous [5,15,16]. Serous tumors are the most common of the epithelial subtypes and comprise two-thirds of all cases [2,3,5,15]. Historically, serous ovarian cancer is classified according to three different three-tiered systems based on morphology/histology. The three systems are the FIGO (the International Federation of Gynecology and Obstetrics) system based on architectural features, the World Health Organization system based on architectural and cytological features, and the Shimizu/Silverberg system based on architectural features, degree of atypical cytological features, and mitotic index, with the most common system being the FIGO system [17]. Within the FIGO system, serous ovarian carcinomas are classified as low grade (Grade 1), intermediate grade (Grade 2), and high grade (Grade 3) [16]. Historically, low grade and high grade serous ovarian tumors were considered to be different grades of the same tumor [5]. However, molecular and genetic studies suggest that it is likely low grade and HGS tumors are distinct diseases with different genetic mutations and different prognoses [5,15,18]. A newer two-tier system combines the current histopathological classification system with molecular genetic findings and clinical features. In this system, ovarian tumors are designated as Type I or Type II [17,19] (Figure 1).

**Figure 2:**
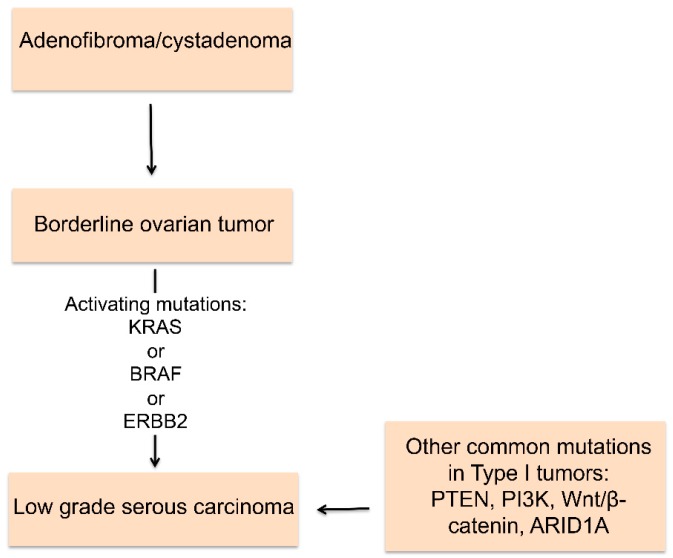
Pathway for Type I tumor formation. Type I tumors appear to form in a stepwise manner from benign precursor lesions. Progression from a borderline ovarian tumors to low grade serous carcinoma commonly includes activating mutations in one of the following members of the MAPK pathway: KRAS, BRAF, or ERBB2.

Low grade serous, mucinous, endometrioid, and clear cell carcinomas fall within the Type I classification [5]. These tumors arise from endometrial tissue, fallopian tube tissue, germ cells, and transitional epithelium [5,14,15,18,21,22]. Type I tumors grow more slowly (are indolent) and are considered to be more genetically stable [5,14,20]. Type II tumors typically have a higher disease volume throughout the peritoneal cavity and a higher incidence of ascites than Type I tumors [20]. They appear to follow a stepwise pattern from a benign precursor to a malignancy with genetic changes in specific cell signaling pathways [2]. Type I tumors are predominantly of non-serous type [10]. Low grade serous ovarian cancer accounts for approximately 5–10% of all serous ovarian cancers [2,10,16]. The most common pathway disrupted in low grade serous ovarian cancer is the mitogen-activated protein kinase (MAPK) pathway [5,6,16,17]. Specifically, activating mutations in BRAF and KRAS are common [2,10,23]. An active MAPK pathway is found in 80% of low grade serous tumors as well as in 78% of their putative precursor lesions (borderline tumors) [16]. Other genes/pathways that are commonly altered in Type I tumors include PTEN, PI3K, ARID1A, Wnt/β-catenin, and ERRB2 [2,6,15,18,20,24,25] (Figure 2).

Prognosis for early-stage tumors is good with a >80% 5-year survival rate with chemotherapy [9]. When dividing all ovarian tumors between stages, Type I tumors are heavily represented in stage I/II (clear cell, 26%; endometrioid, 27%; mucinous, 8%). Only about 36% of early stage tumors are serous [18]. Treatment options for Type I ovarian tumors are identical to those used for Type II tumors and include debulking surgery followed by chemotherapy [17,18]. The response of Type I tumors to chemotherapy is poor due to the relative insensitivity to drug regimens and lack of targeted therapies [7,26]. Low grade serous ovarian tumors have a low response rate to platinum-based therapies with 4% showing a complete response, none with a partial response, 88% with stable disease, and 8% with progression [27]. Another study demonstrated that low grade serous tumors are less responsive than HGS tumors to both paclitaxel (69% vs. 14%) and carboplatin (50% vs. 17%) [27,28]. Type I tumors account for only 10% of ovarian cancer deaths [20]. The poor response of Type I tumors to therapy and the chemoresistance that arises in Type II tumors highlight the need for novel treatment strategies.

HGS tumors comprise 75% of all Type II tumors [3]. HGS neoplasms are typically aggressive and develop rapidly (high mitotic activity) [5,18,20]. Previously, it was thought that HGS ovarian cancer was derived from the ovarian surface epithelium or from cortical inclusion cysts [18,29]. Recent molecular and mouse studies suggest that these tumors likely arise from the epithelium of the distal fallopian tube and that serous tubal intra-epithelial carcinoma (STIC) lesions are the precursors to HGS ovarian cancer [29,30,31]. One study examined histological sections from fallopian tubes of ovarian cancer patients for evidence of STIC lesions. STIC lesions were identified in 61% of the fallopian tubes from HGS patients with 92% of the lesions being in the fimbriated end of the fallopian tube [32]. Kroeger et al. compiled a list of 15 studies showing that approximately 50–60% of HGS tumors are associated with STIC lesions in the fimbriated end of the fallopian tube [3]. Furthermore, in a molecular profiling analysis, HGS tumors with and without STIC lesions exhibited molecular profiles similar to fallopian tube epithelium [29]. To establish if HGS ovarian cancer can be recapitulated in the mouse, transgenic mouse models have been developed. Dicer and PTEN were conditionally deleted in the reproductive tract using anti-Müllerian hormone receptor type 2-directed Cre (*Amhr2-Cre*) [33]. These mice exhibited abnormal proliferation in the stromal compartment of the fallopian tube [33]. Primary and metastatic tumors that developed in the mice were histologically serous carcinoma, and they shared a similar gene expression profile with human HGS tumors [33]. In another model, Pax8-Cre was used to drive the deletion of *Brca/Pten/Tp53* in the fallopian tube. These mice developed STIC lesions and serous carcinomas [31]. Interestingly, loss of PTEN alone in the fallopian tube (via Pax-8-Cre) was sufficient to generate endometrioid and serous borderline tumors [34]. This raises the possibility of fallopian tube origins for some Type I tumors and non-HGS tumors. While it is possible that a portion of HGS tumors arise from the ovarian surface epithelium, it is likely that a major site of origin for HGS tumors is the fallopian tube [30,35].

Unlike Type I tumors, there is a significant amount of genetic instability within the Type II subgroup, and few genes are consistently mutated [5,14]. The main exception is that in Type II tumors, TP53 mutations are common (both inactivating and gain of function) [36,37]. TP53 mutations are rare in Type I tumors [6]. Type II tumors often exhibit active DNA damage repair mechanisms (e.g., PARP) [3,20]. Overexpression of oncogenes ERRB2 (20–67%) and AKT (12–30%) also occur in some cases [6]. Other common mutations in Type II tumors are BRCA1 or BRCA2. Epithelial ovarian cancer is sporadic in 90% of cases with the remaining 10% being hereditary [2]. In 90–95% of hereditary Type II ovarian tumors, there are germline mutations in BRCA1 or BRCA2 [2]. Importantly, BRCA1 and BRCA2 are often mutated or inactivated in spontaneous ovarian cancer. BRCA1 and BRCA2 mutations are detected in around 5–9% and 3–4% of spontaneous ovarian cancer, respectively [38,39,40,41,42]. Loss of BRCA function through other means, particularly promoter methylation, is common in ovarian cancer (particularly when mutations are not present) [43,44]. Therefore, the p53 and BRCA1/2 pathways are highly implicated in development of HGS ovarian cancer.

Most Type II tumors are found in advanced stages of the disease, which leads to a poor overall prognosis. While Type II tumors respond well to chemotherapy (70–80%) initially, almost all patients relapse and Type II tumors result in 90% of all deaths from ovarian cancer [20]. The advanced stage of disease and development of chemoresistance with Type II tumors results in high mortality. A contributing factor to tumor metastasis and chemoresistance is the presence or enrichment of tumor-initiating/cancer stem cells (CSCs) [45]. Devising new treatments that eliminate this cell demographic is of particular interest for HGS ovarian cancer.

## 3. Definition of Ovarian Cancer Stem Cells

**Figure 3:**
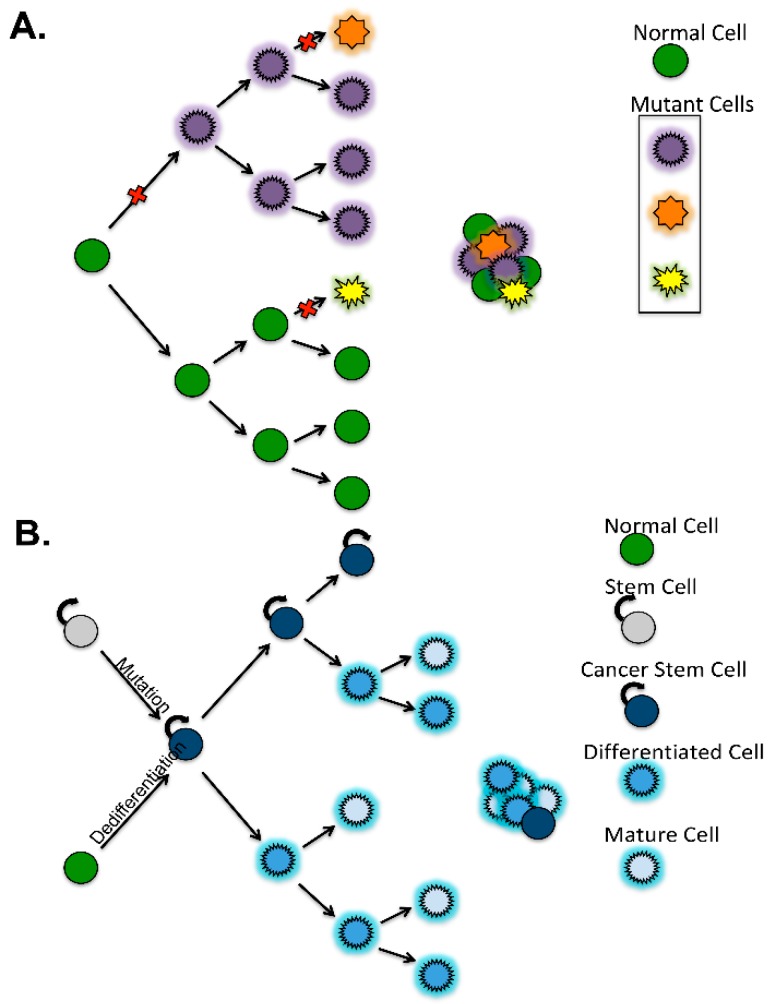
Models of tumor development and heterogeneity. (**A**) The clonal evolution model for tumor initiation. A genetic event occurs in a cell giving rise to a mutant cell population. Any cell is capable of becoming a tumor cell if there is an initiating genetic event. Tumor heterogeneity is due to propagation of cells carrying mutations that are the result of multiple genetic events. (**B**) The cancer stem cell model for tumor initiation. Either a normal stem cell has a genetic event resulting in a cancer stem cell capable of indefinite self-renewal and/or differentiation or a differentiated cell has a genetic event that activates a stem like program within the cell resulting in a cancer stem cell. Tumor cells have a hierarchical inheritance pattern from their cancer stem cell but develop different phenotypes as they acquire further mutations as they differentiate resulting in tumor heterogeneity.

Heterogeneity is a common feature in ovarian cancer tumors. Different models are proposed to explain tumor heterogeneity. In the stochastic or clonal model, tumors arise from a group of homogeneous cells (clonal). Tumor heterogeneity then occurs through random (stochastic) events within this population. Any of the cells within this population can be tumor initiating provided they possess the necessary genetic mutations, epigenetic changes, and a receptive microenvironment [46,47,48,49,50]. The second model (CSC model) recapitulates the stem cell hierarchy found in development of tissues like the hematopoietic system. In this model, tumors are made of groups of heterogeneous cells that all arise from precursor cells with stem-like properties. These “stem-like” precursors differentiate and/or acquire different mutations that lead to diverse activation of pathways. The resultant cells have unique phenotypes and a hierarchical pattern of inheritance from the initiating CSCs [47,49,50,51,52] (Figure 3).

Normal stem cells divide asymmetrically, allowing for self-renewal. One daughter cell retains all the characteristics and programing of the parent cell while the other daughter cell differentiates or acquires new properties [53]. To maintain their undifferentiated state and the ability to self-renew, stem cells reside in a “stem cell niche” comprising various stromal cells, vascular support, and soluble factors that provide a permissive environment [49,54]. CSCs display self-renewal characteristics and retain the ability to produce cells that are highly proliferative and invasive [47,53]. Other characteristics of CSCs include significant DNA repair capability and resistance to therapy [49,53]. In fact, ovarian CSCs (CD133^+^ and Sca1^+^) persisted following chemotherapy in a mouse model of ovarian cancer and in cells treated with carboplatin in vitro [45,55]. Moreover, these cells express stem cell markers and maintain tumor initiating potential [45]. Additionally, in vitro studies demonstrated that treatment of ovarian cancer cells with chemotherapy enriches the stem cell pool [56,57,58]. These studies imply that CSCs are protected from chemotherapy and may be initiators of tumor relapse.

## 4. Stem Cell Identification in Ovarian Cancer

In 2005, Bapat et al. described the first example of a putative ovarian CSC. A single cell was taken from the ascites of an ovarian cancer patient. Once propagated, the cell was able to form anchorage-independent spheroids in culture and was able to seed tumors in mice via serial transplantation over several generations, illustrating the stem-like capabilities of the cell [59]. Since this initial study, many other investigations have been conducted to identify and validate ovarian CSCS. Identification of CSCs relies on the presence of markers (cell surface and intracellular) that are unique to this particular subset of tumor cells [46,47,50]. In ovarian cancer, a variety of markers are used to denote the presence of CSCs. Cells isolated based on these markers can be tested for “stemness” in vitro via spheroid forming assays, resistance to chemotherapeutics, and in vivo with limiting dilution assays (LDAs) to examine the tumorgenicity of the sample [52]. In the LDA, mice are injected with a defined number of cells from a mixed population of cells or cells isolated that express the stem cell markers. The population that is more stem-like will initiate tumors from significantly fewer cells [60]. Table 1 contains a list of some putative ovarian CSC markers.

### 4.1. Side Population

One way in which ovarian CSCs are identified is by their ability to efflux DNA-binding dyes such as Hoechst 33342 and Rhodamine 123 resulting in a side population (SP) using flow cytometry. The ability to efflux these dyes identifies a CSC population that overexpress ATP binding cassette transporters such as MDR1/ABCB1 and ABCG2 that can efflux chemotherapeutic agents [46,49,61,87,88]. This SP demonstrates stem cell properties including the ability to repopulate tumors in an LDA and resistance to chemotherapy. Expression of ABCB1 and ABCG2 correlates with resistance to cisplatin and paclitaxel in ovarian cancer cell lines (2008, KF28, TU-OM-1, OVCAR3, SKOV3) and in cells from patient and mouse ascites [89,90,91]. However, the SP of cells is heterogeneous and can display different combinations of other stem cell markers, so it may be unknown which cells within this population is most “stem-like” or which population(s) are reconstituting the tumor [53].

### 4.2. Cell Surface Markers

Cell surface makers are essential in the identification of CSCs for multiple tumor types. When Bapat et al. first described ovarian CSCs, CD117 was demonstrated to be a cell surface marker for the ovarian CSCs [59]. Human serous ovarian cancer patient-derived xenografts (PDXs) showed that CD117^+^ cells isolated from the xenografts were able to recapitulate a tumor with only 10,000 cells; this was a 100-fold increase in tumor initiating capability compared with the CD117^−^ cells [68]. CD117^+^ cells were also successful at generating tumors when serially transplanted [68]. Other ovarian CSC surface markers include CD24, CD44, EpCAM, and CD133 [13,46,53,61,62]. One of the most commonly reported ovarian CSC markers is CD133. CD133 expression correlates with poor prognosis in ovarian cancer and increased chemoresistance [70,71,72]. In cell lines, CD133 promotes a number of stem characteristics. CD133^+^ and CD133^−^ cells were single cell isolated and expanded from A2780 and PEO1 cell lines [73]. The CD133^−^ cells only produced CD133^−^ cells while CD133^+^ cells divided asymmetrically to produce both CD133^+^ and CD133^−^ cells, suggesting that the CD133^+^ cells retain stem cell properties [73]. CD133^+^ cells exhibit increased resistance to cisplatin and were more tumorigenic in xenograft and serial transplantation studies [73,74]. Another one of the common CSC markers is CD44. CD44 is the hyaluronate receptor and is important in adhesion. In ovarian cancer, CD44 correlates with chemoresistance and tumor progression [63,64,65]. One function of CD44 is to activate Stat3 [66]. CD44 is commonly used as a stem cell marker in combination with CD117, MyD88, E-cadherin/CD34, and CD24/EpCAM. Each of these CD44^+^ cell populations has been demonstrated to have stem-like properties (reviewed in Klemba et al.) [67]. In conclusion, there are multiple surface markers used to identify CSCs in ovarian cancer. Some investigations use these surface markers alone or in combination with other markers. However, we are still uncertain if there is a definitive ovarian CSC marker/population, if multiple CSC populations co-exist, or if CSC identity varies by patient.

### 4.3. ALDH Activity

In addition to cell surface markers, CSCs often are identified using the expression of the enzyme aldehyde dehydrogenase 1 (ALDH1) and its activity. The enzymatic activity of ALDH1 is used to identify and define CSCs in cancer types including breast, colon, liver, and ovarian [46]. Several studies suggest that ALDH1 expression correlates with poor prognosis. In one study of ovarian cancer patients, ALDH1A1 expression was found in 72.9% of tumors, and this expression correlated with decreased progression-free survival (6.05 vs. 13.81 months) [77]. A second study demonstrated that patients with high ALDH1 expression (by immunohistochemistry in >50% of the tumor section) exhibited poorer prognosis [78]. Cell lines with high ALDH1 exhibited increased chemoresistance and tumorgenicity [78]. Silva et al. examined 13 primary human ovarian tumors and 5 ascites samples for various putative CSC markers. ALDH1 was expressed in all cases [75]. Ovarian cancer cell lines were then examined for these CSC markers. Each of the cell lines examined (A2008, SKOV3, HEY-1, A2780, OVCAR8, OVCAR3, and OVCAR432) had a subpopulation of cells with ALDH1 expression [75]. Conversely, knockdown of ALDH1A1 in an orthotopic mouse model (from both taxane- and platinum-resistant cell lines) sensitized the tumors to treatment, resulting in reduced tumor growth [77]. The expression and activity of ALDH1 alone or in combination with cell surface stem cell markers is a popular and accepted method for identifying ovarian CSCs.

### 4.4. Transcription Factors

**Figure 4:**
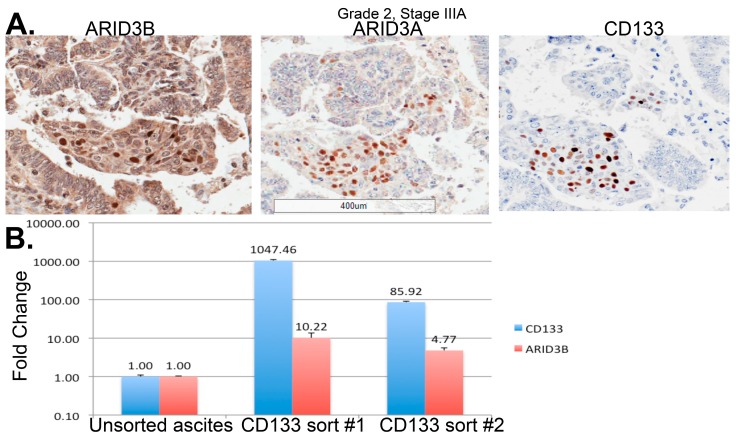
ARID3B expression correlates with CD133-stem cell niche. (**A**) IHC shows nuclear ARID3A and ARID3B co-localize with CD133+ regions in serial HGSOC sections. (**B**) HGSOC patient ascites was sorted for CD133+ cells. RT-qPCR was conducted for Prom1(CD133) and ARID3B on unsorted and independent sorts [98].

Pluripotency transcription factors necessary for normal stem cell maintenance are commonly expressed in ovarian CSCs [53,81,82,83]. In addition to being markers for ovarian CSCs, transcription factors such as OCT4, SOX2, and NANOG are expressed during development and are essential for normal stem cell maintenance and proliferation [62,66,84,92,93,94,95]. Aberrant expression of stem cell genes in differentiated cells, progenitor cells, or stem cell populations can lead to enhanced self-renewal and proliferative capability [96]. Expression of stem cell transcription factors not only provides evidence for the CSC model of tumor development, it also explains in part how stem cell properties of self-renewal and asymmetric division are maintained in CSCs. By comparing normal stem cell populations to CSCs we can gain insight into tumor initiation and regulation of the CSC phenotype. In embryonic stem cells (ESCs) the pluripotency transcription factors form a protein interaction network [83]. Many of these interactions are critical for stem cell functions. In addition, expression of pluripotency factors and protein–protein interactions are retained in CSCs. Among these factors is ARID3B. ARID3B and its paralog ARID3A are expressed in ESCs in a complex with NANOG, OCT4, and NAC1 [83]. ARID3B is overexpressed in serous ovarian cancer and its expression in the nucleus correlates with relapse following chemotherapy [58,97]. ARID3B increases expression of stem cell markers [76]. In particular, ARID3B induces expression of the stem cell marker Prom1 (CD133) [58]. ARID3B additionally increases the pool of CD133^+^ cells, suggesting that it has a role in promoting a stem cell phenotype [58,76]. In fact, ARID3A and ARID3B co-localize with CD133 in ovarian cancer tumor sections. Additionally, ARID3B is enriched in ovarian cancer ascites sorted for CD133^+^ cells (Figure 4). These data suggest that ARID3B^+^ cells are found in a stem cell niche (Figure 4). Future studies on pluripotency factors common in ovarian CSCs including OCT4, MYC, and ARID3B will provide clarity for how cancer stemness is maintained [85,86].

**Figure 5:**
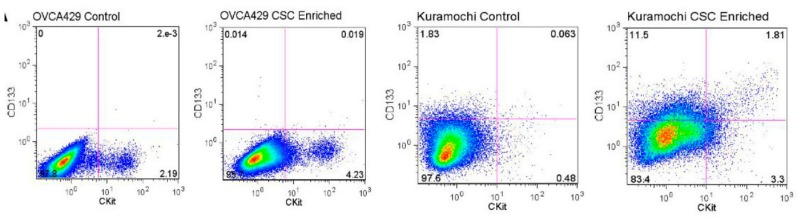
Flow cytometry for the stem cell markers CD117 and CD133 on ovarian cancer cells before and after CSC enrichment. Untreated OVCA429 and Kuramochi cells or cells enriched for CSCs (by treatment with cisplatin and paclitaxel followed by culturing CSCs in stem cell media on ultra low adhesion plates) [56] were stained for stem cell markers CD117 (cKIT is the gene that encodes CD117) (X-axis) and CD133 (Y-axis).

Different stem cell markers may confer different selective advantages to different pools of “CSCs”. Patients may have more than one pool of stem cells and different patients may have CSCs with different phenotypes. An example is included in Figure 5. To enrich for CSCs, OVCA429 and Kuramochi cells were untreated or treated with cisplatin and paclitaxel and then cultured on nonadherent plates in stem cell media [56]. Flow cytometry was performed for CD117 (gene = CKIT) and CD133. OVCA429 cells have a clear CD117^+^CD133^−^ population of CSCs that is enriched following chemotherapy treatment. Following chemotherapy treatment, multiple cell populations are expanded in Kuramochi cells including CD133^+^/CD117^−^, CD133^+^/CD117^+^, and CD117^+^/CD133^−^. These experiments suggest that different stem cell pools may be more prevalent in an individual cell type or patient tumor. Importantly, each of the CSC markers may have its own each unique function. The kinase activity of CD117 may provide a survival advantage over CD117^−^ cells [69]. However, CD133^+^ cells may have an adhesion or metastatic advantage over cells lacking CD133 [76]. Although we can detect cell-to-cell variation in the expression of markers, we do not know if these different CSC lineages arise from common progenitors. CSC lineage tracing to define the hierarchy of cells in a stem cell population has not been conducted for all putative ovarian CSC subtypes. Additionally, LDAs need to be conducted to verify stem cell potential for each putative ovarian CSC population. In order for studies of CSCs to be translational, we will need to define how the different CSC populations pertain to patient prognosis, relapse, and response to therapy. Moving forward, we need to establish the clinical significance of different ovarian CSC marker profiles [47,52,53,61,99]. Comparing survival and relapse potential for patients based on these different marker profiles is essential for us to develop effective treatments for the clinically relevant ovarian CSC populations.

## 5. Pathways That Promote Stemness and Chemoresistance in HGSOC

We chose to focus on the major pathways that drive both stemness and chemoresistance in HGS ovarian cancer. These properties of highly metastatic HGS ovarian cancer are inextricably linked. Understanding the pathways that are most pertinent to metastatic HGS ovarian cancer will provide us with putative targets to develop efficacious therapeutic agents. As there are numerous pathways involved in stemness and chemoresistance, we will highlight the ones that have a clear role in ovarian cancer and are potentially targetable.

### 5.1. PI3K/PTEN/AKT Signaling

Aberrant PI3K/PTEN/AKT signaling often results from genomic alterations in many cancers including clear cell ovarian cancer. In HGS carcinoma, there are few mutations in the components of the PI3K/PTEN/AKT pathway, but by immunohistochemistry (IHC) about half of the HGS tumors have evidence of pathway activation [100,101]. A meta-analysis of the literature reports that both univariate and multivariate analysis show that high expression of activated AKT (pAKT) is associated with poor progression-free survival and poor overall survival [102]. Due to mutations in many parts of the PI3K/PTEN/AKT pathway, activated AKT signaling is highly relevant for ovarian cancer development and progression.

The PI3K/PTEN/AKT pathway is also implicated in ovarian CSCs. PI3K/PTEN/AKT signaling regulates enrichment of CSCs, maintenance of a CSC phenotype, and chemoresistance [103,104,105,106]. Spheroids derived from SKOV3 and HO8910 cell lines expressed elevated phosphorylated AKT1 and decreased expression of PTEN [103]. The spheroids exhibited increased resistance to paclitaxel [103]. Conversely, inhibiting AKT1 activation decreased spheroid formation and migration [104]. Knockdown of AKT1 via siRNA resulted in the loss of CSC marker expression (OCT4, SOX2, ALDH1, and ABCG2) as well as loss of spheroid formation and paclitaxel resistance [104]. These studies demonstrate the importance of the PI3K/PTEN/AKT pathway in CSC formation, maintenance, and chemoresistance to paclitaxel.

The PI3K/PTEN/AKT pathway also regulates cisplatin resistance in ovarian cancer. In cisplatin-resistant A2780 cells (A2780-CP), AKT regulates the expression of PPM1D [105]. PPM1D inhibits the DNA damage and apoptotic response after DNA damage occurs [105]. Downregulation of AKT activity results in loss of PPM1D stability and increases its degradation [105]. Loss of PPM1D increases the response of the A2780-CP cells to cisplatin [105].

The PI3K/PTEN/AKT signaling pathway promotes the enrichment of ovarian CSC populations and regulates ovarian CSC chemoresistance, thus making it an ideal target for therapeutics to eliminate ovarian CSCs. There are currently PI3K/PTEN/AKT inhibitors such as BKM120, Everdimus, and Perifosine that are being used to treat cancer patients [100]. Future efforts to stratify patients that are likely to benefit from PI3K/PTEN/AKT inhibition will be needed for this therapy to be effective in ovarian cancer patients.

### 5.2. Jak2/STAT3

Proliferation, survival, and differentiation are all regulated by the Jak2/STAT3 pathway in several solid tumors [107]. In ovarian cancer, the Jak/STAT pathway is constitutively active in most cases [108]. Jak/STAT is implicated for having a key role in the development of HGS ovarian cancer. Activation of STAT3 via phosphorylation at Tyr705 and the loss of the STAT3 inhibitor PIAS3 may serve as a tumor-initiating event in the distal fallopian tube for the formation of HGS ovarian cancer [109]. Phosphorylated STAT3 is expressed in 86% of ovarian tumors examined (from different histotypes) and constitutive pSTAT3 expression is expressed in 63% of the HGS tumors examined [110]. Phosphorylated, nuclear STAT3 is associated with poor prognosis [110]. In tissue microarrays (TMAs), patients whose tumors had high nuclear pSTAT3 staining (>10% nuclei stained) had poorer survival rates than women with low nuclear pSTAT3 staining (<10% nuclei stained) [110]. These patient findings implicate the Jak/STAT pathway as being highly important for ovarian cancer initiation and progression.

The Jak/STAT pathway also regulates ovarian CSCs. CD24^+^ ovarian CSCs require Jak2/STAT3 signaling for growth and metastasis [111]. Primary tumors generated in the *Apc^−^*; *Pten^−^*; *Trp53*^−^ (transgenic mouse model in which APC, PTEN, and Trp53 are conditionally deleted in the ovarian surface epithelium) were collected, dissociated, and sorted via fluorescence-activated cell sorting (FACS) using stem cell markers [111]. LDAs confirmed that the CD24^+^ cells isolated were a CSC population [111]. This population of cells expressed elevated pSTAT3 and stem cell marker NANOG, which is required for stem cell renewal [111]. CD24^+^ cells were injected into mice and the mice were then treated with cisplatin or with cisplatin+TG101209, a Jak2 inhibitor [111]. The mice treated with cisplatin+TG101209 showed significantly increased survival and almost no metastases (1 out of 14) [111].

Other studies show a role for the Jak/STAT pathway in ovarian CSC maintenance and chemoresistance. Abubaker et al. collected tumor cells from patient ascites or the HEY8 ovarian cancer cell line and treated them with paclitaxel [108]. Treatment with paclitaxel induced the expression of CSC markers CD117, OCT4, and EpCAM in ascites and HEY8 cells [108]. In both the paclitaxel-treated ascites and HEY8 cells, the Jak2/STAT3 pathway was activated [108]. This suggests that the Jak2/STAT3 pathway regulates the expression of stem-like genes necessary for CSC maintenance. Moreover, paclitaxel-treated cells were also treated with the Jak2-specific small molecule inhibitor (CYT387), which resulted in inhibition of the Jak2/STAT3 pathway activation, loss of stem cell marker expression, and increased sensitivity of the cells to paclitaxel treatment [108]. When paclitaxel-treated and paclitaxel+CYT387-treated cells were injected into mice, the mice injected with the paclitaxel+CYT387-treated cells showed a reduced tumor burden and enhanced sensitivity to paclitaxel [108]. These studies demonstrate that in models of ovarian cancer, Jak2 inhibitors are effective at reducing stem cell characteristics and inhibiting tumor growth. These inhibitors also increase survival and response to therapy. Because the Jak/STAT pathway promotes stemness and chemoresistance in the CSC population, it is a viable target for therapies aimed at reducing ovarian CSC populations.

### 5.3. NFκB

The NFκB pathway plays a role in normal cellular processes such as survival, proliferation, and apoptosis. In cancer the NFκB pathway is implicated in invasion and metastasis. However, the pathway is also involved in CSC maintenance [112]. In ovarian cancer, both the canonical and noncanonical NFκB pathways are active. A CD44^+^ ovarian CSC population isolated from patient ascites exhibited constitutive NFκB pathway activation via a luciferase reporter assay, formed spheroids in culture, and formed tumors when injected into mice [13]. Another study showed that CD44^+^ CSCs from SKOV3 cells (that also express NANOG, SOX2, and OCT4) exhibited increased expression of NFκB pathway members RelA, RelB, and IKKα [113]. Inhibition of the NFκB pathway with a dominant-negative form of IκBα resulted in a decrease in the CD44^+^ CSC population with a reduction from 65.3% CD44^+^ cells to just 27.7% [113]. These data suggest that NFκB signaling regulates expression of stemness genes.

The NFκB pathway is also involved in ovarian CSC chemoresistance. CD44^+^ ovarian CSCs from patient ascites have constitutively active NFkB [13]. When treated with TNFα, the CD44^+^ cells showed increased NFκB activity and cytokine production as well as resistance to TNFα-induced apoptosis [13]. The resistance to apoptotic pathway activation suggests a mechanism for ovarian CSC survival when treated with chemotherapeutics. Treatment of ovarian CSCs with Eriocalyxin B (EriB) inhibits the NFκB pathway and induces cell death in ovarian CSCs [114]. EriB inhibited the TNFα-induced NFκB activity and cytokine production and sensitized the cells to TNFα- and FasL-induced cell death [114]. This suggests that inhibition of the canonical NFκB pathway could sensitize ovarian CSCs to therapy [114].

While many studies focused on the canonical NFκB pathway, the noncanonical pathway is also active in promoting stemness and chemoresistance in ovarian cancer. RelB in particular is important for ovarian CSC regulation. RelB is overexpressed in ovarian CSC populations including CD44^+^ SKOV3 cells and ALDH^+^/CD133^+^ OV90 and ACI23 cell lines [113,115]. In the OV90 and ACI23 cells, ALDH1 activity and expression of RelB both increase with carboplatin treatment [115]. This suggests a role for the noncanonical NFκB pathway and RelB in promoting stemness and chemoresistance. Knockdown of RelB with shRNA reduced the number of ALDH^+^/CD133^+^ CSCs in vitro in both cell lines and in xenografts by 50% [115]. The RelB knockdown decreased expression of other stem cell markers (NANOG and CD44) and increased sensitivity to carboplatin [115]. In addition, ACI23 and OV90 cells, when stably transfected with inducible shRNA for RelB, showed reduced spheroid formation and reduced tumorgenicity [115]. The noncanonical pathway through RelB promotes tumor growth as well as the expression of stemness genes [115]. RelB also regulates chemoresistance in ovarian CSCs [115]. Thus, both the canonical and noncanonical NFκB pathways are excellent targets for therapeutics to reduce the CSC population.

### 5.4. Notch

Notch signaling has a role in multiple cellular processes. Notch is a critical component in regulating progenitor cell maintenance, differentiation, cell proliferation, and apoptosis. Notch is also important for cell–cell communication [116,117]. In HGS ovarian cancer, Notch3 expression is amplified/overexpressed [118]. By analyzing 31 fresh HGS ovarian cancer samples, Notch3 amplification correlated with protein expression [118]. Notch3 was overexpressed more often in high grade tumors (66%) than in low grade tumors (33%) [118]. Further, according to The Cancer Genome Atlas (TCGA), Notch3 is amplified in 17% of HGS tumors. The most highly expressed Notch3 ligand in ovarian serous carcinoma is Jagged 1, which is predominantly expressed in the mesothelial cells within the tumor microenvironment, suggesting a role for Notch3/Jagged 1 signaling in cell adhesion and proliferation [119].

In the majority of patients with recurrent HGS ovarian cancer, Notch3 is overexpressed [120]. Tumors from patients with either primary disease or recurrent disease were examined for Notch3 overexpression and survival [120]. In the group with primary disease, there was no difference in survival between those with Notch3 overexpression and those without [120]. Those in the group with recurrent disease did show a difference. Those expressing high Notch3 levels had decreased overall survival (22 vs. 37 months) and decreased progression-free survival (3 vs. 8 months) suggesting that Notch3 expression is a factor in the recurrence of ovarian cancer as well as a prognostic indicator in recurrent disease [120].

Chemoresistance is a hallmark of CSCs and disease recurrence/relapse, and Notch3 expression affects the expression of stemness factors as well as chemoresistance. The transcription factor OCT4 promotes self-renewal of ovarian CSCs while SOX2 is required for their maintenance [84,92]. Overexpression of Notch3 in ovarian cancer cell lines (IOSE-80pc and MPSC1) enhances expression of stem cell markers (NANOG, OCT4, and SOX2) and increases expression of the ABCB1 transporter protein [120]. The ABCB1 transporter increases chemoresistance in these ovarian CSCs and NANOG promotes the epithelial to mesenchymal transition (EMT) in ovarian cancer [121]. To demonstrate the role of Notch3 on chemoresistance, Nocth3 was knocked down in OVCAR3 cells using shRNA resulting in reduced IC_50_ compared to control cells [120]. These studies all implicate Notch3 signaling in ovarian CSC chemoresistance.

Other Notch signaling molecules are also implicated in stemness and chemoresistance including Jagged 1 and downstream signaling molecules. Downregulation of Jagged 1 in SKOV3TRip2 cells via siRNA increased sensitivity of cells to docetaxel [122]. In ovarian cancer cells isolated for the SP, Notch pathway genes (FPTG, ST3GAL6, and ADAM19), stem cell markers NANOG and OCT4, and three ABC transporter genes (ABCG2 [both lines], ABCC4 [SKOV3 only], and ABCB1 [A224 only]) were induced [95]. Collectively, the data suggest that Notch signaling is involved in promoting stemness and chemoresistance, and expression of Notch3 in particular may serve as a prognostic indicator for patients with recurrent disease. Notch signaling is an attractive target for therapeutics aimed at ovarian CSCs. Currently, there are experimental γ-secretase inhibitors, γ-secretase modifiers, Notch soluble decoys, and negative regulatory region monoclonal antibodies that are already being developed [116].

### 5.5. Wnt

**Figure 6:**
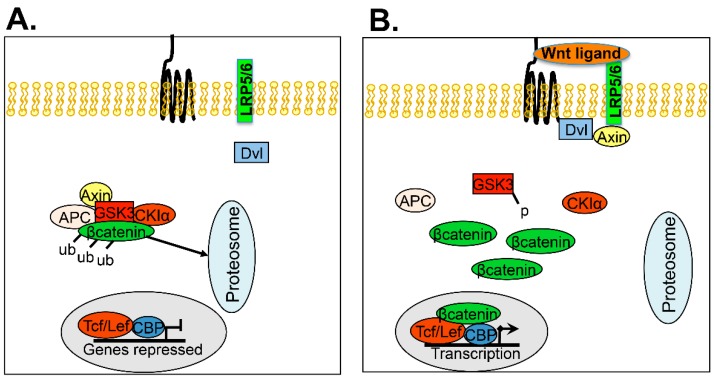
Wnt Signaling Cascade. (**A**) Basal state without the presence of Wnt ligand activation. β-catenin is ubiquitinated and sent to the proteosome for destruction. (**B**) Activation of the Wnt pathway via binding of a Wnt ligand to the Frizzled receptor and LRP5/6 resulting in recruitment of Disheveled (Dvl) and axin to the cell membrane. β-catenin is released from the destruction complex and translocates to the nucleus to act as a co-transcription factor.

Wnt signaling is particularly important during development where it regulates cell fate determination during embryogenesis including the cardiovascular system, central nervous system, and craniofacial development [116,123]. In adults, Wnt signaling is critical for self-renewal in tissues (e.g., bone growth plate, hair follicles, colon, etc.) [116,124,125]. The major processes regulated by noncanonical Wnt signaling include cell polarity and motility; however, Wnt also plays a role in maintaining stem cells, quiescence, and chemoresistance [126]. Wnt signaling is complex and many components of Wnt signaling are implicated in ovarian CSCs and chemoresistance (Figure 6).

With regards to ovarian cancer, Wnt signaling is involved in normal development of the ovarian and fallopian tube stem cells. Wnt signaling also has functions in tumor development. LGR5 is a stem cell marker for ovarian stem cells and LGR6 is a stem cell marker for the fallopian tube, and expression of either one is a sign of elevated Wnt signaling [127,128,129]. LGR5 and LGR6 are expressed in HGS tumors [127]. LGR5^+^ cell-driven lineage tracing was performed in mice, illustrating the importance of LGR5 and Wnt signaling in embryonic and adult ovarian stem cells for homeostasis and regenerative repair and self-renewal [130]. Since the fimbria of the fallopian tube are implicated as a site of origin in HGS tumors, fallopian tube stems cells also must be examined [129]. Using a Tcf-eGFP reporter and confocal microscopy on fallopian tube organoid cultures, active Wnt signaling was needed for the expression of stem cell factors to support organoid growth [129]. Understanding how abnormal regulation of Wnt signaling drives initiation or maintenance of ovarian CSCs is critical.

Disregulation of Wnt signaling is frequently involved in the development of cancer [123,131]. In ovarian cancer, aberrant Wnt signaling differs by histotype. Wnt signaling stabilization and subsequent nuclear translocation of β-catenin leads to activation of Wnt target genes including those involved in stemness. β-catenin is frequently mutated at GSK3β phosphorylation sites that allow β-catenin to be ubiquinated and degraded in the absence of Wnt signaling (54%) resulting in nuclear localization in approximately 70% of cases of low grade endometrioid ovarian carcinomas [132]. Activating mutations of proteins in the Wnt pathway are rare in serous ovarian carcinomas [132]. However, there is evidence of nuclear β-catenin in HGS [132]. With regards to the noncanonical Wnt pathway, Wnt5A was highly expressed in a collection of 583 ovarian tumors and it is found in the ascites [126,132]. Receptor tyrosine kinase-like orphan receptor 1 (ROR1) (a pseudokinase and receptor for Wnt5A) is expressed in ovarian cancer and is correlated with poor outcomes [79]. Survival analysis showed that patients with high expression of ROR1 had significantly reduced progression-free survival and overall survival [79]. Cells isolated from ROR1^+^ patient-derived xenografts exhibited stem-like qualities including ALDH1 expression, ability to form spheroids, and increased tumorgenicity [80]. These data suggest that ROR1 is a potential CSC marker for ovarian cancer and that noncanonical Wnt signaling is a component of ovarian cancer stemness.

In ovarian CSCs, Wnt signaling helps promote both stemness and chemoresistance. The CSC marker/receptor tyrosine kinase, CD117, is upregulated in ovarian CSCs. Many factors contribute to acquisition of CD117 expression including the hypoxic microenvironment of the stem cell niche [106]. CD117 leads to activation of AKT and the phosphorylation of GSK3β and nuclear expression of β-catenin [106]. β-catenin activity induces expression of ABCG2, a drug transporter which increases cisplatin and paclitaxel resistance [106]. Therefore, the hypoxic niche supports stemness by activation of Wnt target genes.

Wnt signaling in ovarian cancer CSCs is complex. Collectively, the patient studies combined with cell culture and animal models suggest that multiple Wnt signaling pathways contribute to stemness and chemoresistance in ovarian cancer. A number of potential molecules in the Wnt pathways may be viable targets for therapeutic intervention. Wnt inhibitors such as compounds that target Disheveled (NSC668036 and FJ9), Frizzled receptor antibody, Thiazolidinedione (target β-catenin reverse transport), and Sulindac (unknown action but potentially effects β-catenin proteasomal degradation) are being examined for use in cancer treatment [116]. Deciphering the cross-talk between Wnt and other pathways in addition to more sophisticated assessment of the contribution of particular Wnt molecules and pathways will enable development of future Wnt-targeted drugs that can be used in ovarian cancer treatment.

### 5.6. Hedgehog

During embryogenesis, Hedgehog signaling (Hh) regulates tissue polarity as well as patterning and stem cell maintenance [116]. In cancer, the Hh pathway is dysregulated in one of two ways: (1) constitutive expression of endogenous ligand (e.g., Sonic hedgehog [Shh]) or (2) mutations of proteins within the pathway (Patched, SMO, SUFU) [133]. We will explore the ways Hedgehog signaling has emerged as an important regulator of proliferation, chemoresistance, and stemness in ovarian cancer [133,134].

Overexpression of Gli1 (a transcription factor activated by Hh signaling) as well as PTCH (Hh receptor) is correlated with poor prognosis and survival in patients [133]. Eighty cases of epithelial ovarian tumor were examined by IHC [133]. All cases expressed PTCH, though PTCH was highly expressed in 34.1% of cases [133]. Gli1 expression varied by histotype of the tumor with high Gli1 expression being most common in serous tumors [133]. High expression of either Gli1 or PTCH correlated with poor survival compared to those patients with low expression [133]. These data suggest that Gli1 and/or PTCH expression may be prognostic indicators for ovarian cancer patients. Gli1 antagonists such as HPI 1–4 that are currently being developed as well as drugs targeting PTCH may be useful therapies for ovarian cancer patients with activated Hh signaling.

In ovarian cancer, Gli1 appears to be a critical contributor. Gli1 is a regulator of proliferation and tumor growth in ovarian cancer. Gli1 is elevated in several ovarian cancer cell lines (OVCAR5, OV-202, and OV-167) compared with normal ovarian surface epithelium [135]. Inhibition of the Hh pathway with cyclopamine resulted in Gli1 decreasing in a dose-dependent manner (60–80%) [135]. The decrease in Gli1 mRNA and protein correlated with a decrease in proliferation in all three cancer lines [135]. In addition to the in vitro results, a mouse xenograft model using OVCAR5 cells found that cyclopamine significantly inhibited tumor growth [135]. In agreement with these findings, exogenous expression of Gli1 in ovarian cancer cell lines SKOV3, OVCAR3, and OVCA433 increased cell proliferation 2-fold and increased invasiveness 200–500% over control; whereas knockdown of Gli1 with siRNA suppressed proliferation and invasiveness (40–60%) [133]. These studies suggest that Gli1 is an important regulator of proliferation and tumor growth in ovarian cancer.

The Hh pathway regulates stemness in ovarian cancer. In one study, ES2, SKOV3, and TOV112D cells were treated with recombinant Shh and Ihh, both Hh pathway agonists [134]. In all three cell lines, spheroid formation increased significantly [134]. When treated with cyclopamine, there was significant impairment of spheroid formation [134]. This demonstrates a role for the Hh pathway in maintaining stemness in ovarian cancer.

Gli1 also is implicated in chemoresistance in ovarian cancer cells. Gli1 has an interesting role in the DNA damage response following cisplatin treatment [136]. In cisplatin-resistant A2780 cells (A2780-CP), cells with anti-Gli1 shRNA or a scrambled shRNA were treated with cisplatin and then DNA repair was assessed [136]. After 12 h the control cells had repaired 78% of the DNA adducts compared to 33% in cells treated with anti-Gli1 shRNA [136]. In addition to impairing the cell’s ability to repair the cisplatin adducts, pretreatment with the anti-Gli1 shRNA sensitized the cells to cisplatin resulting, in a shift of the IC_50_ from 30 μM to 5 μM [136]. This suggests that Gli1 regulates DNA adduct repair and sensitivity to cisplatin in ovarian cancer. Additionally, Gli1, SMO, and PTCH are overexpressed in borderline and malignant ovarian cancer [137]. Moreover, Gli1 and SMO were highly overexpressed in platinum-resistant ovarian cancer [137]. Both cell culture and patient studies suggest an important role for Gli1 and Hh signaling in ovarian cancer chemoresistance.

While Hh signaling is studied in regard to other cancer types, Hh signaling in ovarian cancer is relatively understudied. Current findings suggest that Gli1 has an important role in ovarian cancer stemness, tumorigenicity, and chemoresistance. Further studies on the role of Hh signaling in ovarian cancer will allow for personalized medicine approaches for those patients with active Hh. Future therapy options could include the Hh inhibitor GDC-0449 that is currently in clinical trials for use in ovarian cancer [138].

### 5.7. Developing Therapeutics Targeting Ovarian Cancer Stem Cells

There are multiple pathways involved in promoting a stem cell phenotype and chemoresistance in ovarian cancer. Each pathway has the potential to be therapeutically targeted. However, a major challenge is defining which population of cells needs to be targeted with pathway inhibitors.

If a therapeutic goal is to eliminate the CSC population, more studies are needed to define CSC populations, markers, and critical pathways that are required for stem cell maintenance (Table 2: Summary of targetable genes).

## 6. Future Studies

Ovarian CSCs in HGS ovarian cancer are an attractive target for therapeutics in order to prevent relapse following chemotherapy. Prior to targeting these insidious cells, a number of issues should be considered. One complication in treating patients with HGS ovarian cancer is the amount of heterogeneity found within the tumors. Additionally, HGS is characterized by genomic instability rather than specific driving mutations. This level of heterogeneity makes identifying drug targets that help a wide population of HGS ovarian cancer patients difficult. More phenotypic, genetic, and epigenetic studies of patient CSCs need to be conducted to assess which CSC populations are the most critical ones to target. Hierarchical lineage tracing efforts will allow us to decipher if different CSC populations arise from a common progenitor cell. Detailing the mechanisms that are required for CSC maintenance is critical. Delineating the role of the microenvironment in CSC maintenance is also important. Do these varying marker profiles denote differing niches for the CSCs and, therefore, different survival and renewal pathways that are active in different populations of CSCs? Are different CSC subpopulations present at different times during cancer progression? These questions underscore the need for personalized medicine in the treatment of ovarian cancer. Three potential targets for new therapeutics include stem cell markers, stem cell signaling pathways needed for renewal and/or survival, and the stem cell niche. Careful studies examining the contribution of CSC subpopulations and signaling pathways to CSC survival and maintenance will lead to directed therapeutic target design.

## Figures and Tables

**Table 1:** Putative Ovarian Cancer Stem Cell Markers.

Marker	Type of Protein	Suspected Role in Stem Cells	References
CD24	Cell surface transmembrane glycoprotein	Stem gene expression, tumor initiation, chemoresistance, stem cell maintenance	[46,53,61,62]
CD44	Cell surface transmembrane glycoprotein (hyaluronic acid receptor)	Chemoresistance, tumor initiation, stem gene expression, spheroid formation	[13,46,53,61,62,63,64,65,66,67]
cKit/CD117	Tyrosine kinase receptor	Chemoresistance, stem cell maintenance, tumor initiation	[11,53,59,61,68,69]
PROM1/CD133	Cell surface transmembrane glycoprotein	Tumor initiation, chemoresistance, spheroid formation, high cell proliferation	[13,46,53,61,62,70,71,72,73,74,75,76]
ALDH1	Cytosolic aldehyde dehydrogenase enzyme	Tumor initiation, chemoresistance, spheroid formation	[46,53,61,75,77,78]
ROR1	Tyrosine kinase receptor	Spheroid formation, tumor initiation, proliferation	[79,80]
SOX2	Transcription factor	Stem cell maintenance, self-renewal	[8,81,82,83,84]
NANOG	Transcription factor	Stem cell maintenance, self-renewal, chemoresistance	[8,53,61,66,81,82,83]
POU5F1/OCT4	Transcription factor	Tumor initiation, chemoresistance	[8,53,61,81,82,83]
MYC	Transcription factor	Tumor initiation, chemoresistance	[85,86]
EpCAM	Cell surface membrane glycoprotein	Tumor initiation, spheroid formation, proliferation	[13,46,53,61,62]
MDR1/ABCB1	ATP binding cassette transporter	Chemoresistance	[46,49,53,61,66,87,88,89,90,91]
ABCG2	ATP binding cassette transporter	Chemoresistance	[46,49,53,61,87,88,90,91]

**Table 2:** Summary of targetable genes.

Pathway	Gene	Potential Therapeutics in Trials
**PI3K/PTEN/AKT**	AKT1	BKM120, Everdimus, Perifosine
	PTEN	
	PPMID	
**Jak/STAT**	STAT3	
	JAK2	
**NFκB**	RelA	
	RelB	
	IKK	
	IκBα	
	TNFα	
**Notch**	Notch3	γ-secretase inhibitors, γ-secretase modifiers, Notch soluble decoys, negative regulatory region monoclonal antibodies
	Jagged1	
**Wnt**	β-catenin	NSC668036, FJ9, Frizzled receptor antibodies, Thiazoldinedone, Suldinac
	Wnt5A	
	Disheveled	
	Frizzled	
**Hedgehog**	Patched	HPI-1, HPI-2, HPI-3, HPI-4, GDC-0449
	Gli1	
